# The effects of perceived discrimination, social support and ethnic identity on mental health of immigrant adolescents

**DOI:** 10.21307/sjcapp-2021-014

**Published:** 2021-06-24

**Authors:** Derya Atalan Ergin

**Affiliations:** Ministry of Education, Ankara, Turkey

**Keywords:** Immigrant, adolescent, perceived discrimination, social support, ethnic identity, mental health

## Abstract

**Background**: The number of immigrants has been increasing. Immigrant adolescents experience an acculturation process that affects particularly their ethnic identity, perceived discrimination, and relationships with their peers, which would have significant impact on their mental health. The ethnic composition of social environments might affect this relationship.

**Objective:** The main purpose of the current research is to examine the effect of peer attachment, social support, ethnic identity, and perceived discrimination on immigrant adolescents’ mental health.

**Method:** The sample included 226 Syrian immigrants (X^¯^_age_ = 13.31, SD=1.67, 70.8 % girls). Adolescents live in a homogenous social environment where proportion of Syrian is higher. Two hierarchical regression models were used to predict depression and emotional problems. In both models, the predictive roles of social and psychological factors were examined in separate steps.

**Results:** The regression analysis results for depression emphasized peer attachment, social support, and ethnic identity did not affect the depression after controlling the effect of emotional problems. Similarly, regression analysis results for emotional problems showed that peer attachment, social support, and ethnic identity did not affect depression after controlling the effect of emotional problems. The results also revealed that perceived discrimination was a risk factor for both depression and emotional problems.

**Conclusions:** The results underlined the importance of psychological variables on immigrant adolescents’ depression. Past research emphasized that ethnic identity and peer support had a buffering effect on mental health. The current study participants were living in a different area where they attended schools for only immigrants. The social environment was totally different from the host culture. These reasons may account for why social support from ethnic peers and ethnic identity development did not emerge as a protective factor in the present study. The results will further be discussed in terms of the importance of interaction between ethnic and host culture.

The number of immigrants has been increasing recently. According to the World Migration Report (1), the number of immigrants is 271,642,105 in the world; fourteen percent of this number was under the age of 20. The immigration rate from Syria is higher than in most other countries due to long-term conflict-induced displacement (1). Turkey is one of the world’s largest host countries that Syrians constitute a large proportion of immigrants with 3,639,572 (1, 2). 1,728,540 (47.4%) of these people are children between 0-18 (2). These immigrants have to cope with both pre-migration stressors like the effect of war (3) and post-migration issues like acculturation (4). The migration and its accompanying stressors may relate to their mental health problems (5).

Turkey hosted 677,917 Syrian adolescents in the 10-14 age range. Adolescence is a lifespan in which requires many developmental tasks. This period can be more complicated for immigrant youth facing migration difficulties. Acculturation influences developmental processes. Developmental contextualism is based on the defining idea of an interaction between the organism and the context (6). Researches examining this interaction showed that acculturation is associated with psychological problems (7), ethnic identity development (8), peer acceptance (9), and peer attachment (10). Immigrant adolescents face an acculturation process that impacts their ethnic identity and peer relationships associated with their mental health.

## Mental health

Different types of traumatic events (e.g., bereavement, neglect, school violence) affect adolescents’ mental health (11). The forced migration process is a traumatic event that might be riskier with bereavement, economic loss, cultural shock, and conflict. The result of a meta-analysis emphasized downward mobility or underemployment because migration is related to negative mental health (12). Immigrant adolescents’ researches showed that migration was related to many types of negative mental health problems such as schizophrenia (13), anxiety (14), PTSD, and depression (15). Gutmann et al. (16) revealed that internalizing and externalizing problems were higher among immigrant adolescents than their native peers. The research with war-affected immigrant adolescents in Turkey emphasized a higher level of mental problems like post-traumatic stress disorder (17). It specified that the most significant factor on Syrian adolescents’ mental health is the loss of loved ones in pre-migration and the loss of culture and support in post-migration these (18). The post-migration process and accompanying issues were examined in this research.

Acculturation is a process of change that results from contact between groups and individuals of different cultures (19). Berry (4) defined four types of acculturation: integration, assimilation, separation, and marginalization. In both assimilation and marginalization, individuals are not attached to either the host or ethnic culture. Researches have shown that these two statuses were related to negative mental health outcomes (21, 22). On the other hand, attachment to cultural identity is related to a lower level of depression (23), better well-being (24), low social anxiety (22), and better mental health (25). Understanding the immigrants’ acculturation process is critical in delivering health care and other services to them (26).

## Perceived discrimination

It is suggested that migration research underscore the importance of taking into account the stressful social conditions of everyday life because migration can be related to mental health problems (27). Perceived discrimination is broadly defined as an individual’s perception of being treated unfairly by other people due to personal attributes such as race, ethnicity, age, gender, socioeconomic status, weight, sexual orientation, or other characteristics (28). It has impacted the mental health of ethnic minorities as a chronic stressor (29). Research has shown that perceived discrimination is associated with the emergence of depressive symptoms (30, 31), lower well-being (32) and life satisfaction (33), higher levels of violent behavior (34), alienation, and risky behaviors (35). Besides, the research has shown that increased acculturation is associated with decreased perceived discrimination (36-38). Therefore, it is critical to consider the relationship between them due to their importance in the acculturation process.

## Peer relationship: attachment and support

Adolescence is a critical life period in developing peer relationships (39). Peer relationships might be more important for immigrant adolescents during the acculturation process. They are separated from their social network, which decreases their accustomed level of support (40). Therefore, peer relationships should also be evaluated for contributing to the acculturation process for immigrant adolescents. The research investigated the peer relationship with different heading such as quality of the relationship (41), peer dynamics, peer popularity, and deviant peer affiliation (42), or peer connectedness (43). Peer attachment and peer support related to positive mental health outcomes (44, 45) are usually used to focus on peer relationships' adaptation dimension (46).

Social support and attachment are crucial for mental health and acculturation (47-50). For example, it is reported that social support from peers has a positive relationship with health-related quality of life (51), mental health (47), self-esteem (52), less internalizing problems (53), and social anxiety (54) among immigrant adolescents. Similarly, peer attachment has negative correlations with depressive symptoms and positive correlations with a sense of coherence (48). Moreover, both peer social support and peer attachment contribute to the acculturation process’ success (50, 55, 56). High levels of social relations with peers having the same ethnic origin might be opening up opportunities to behave by one’s cultural habits. This might increase discordance with the host culture and reduce acculturative stress (27, 57). It specified that social environments’ ethnic composition affect interpersonal relationships (58-61). In this research, data were obtained from immigrant-dense social environments, contrary to researches mostly obtained from immigrant-sparse environments.

## Ethnic identity

Identity formation is an essential developmental task during adolescence. Thus, immigrant adolescents might experience a conflict between ethnic and host cultural identity. Ethnic identity is defined as individuals’ degree of belongingness and feelings about the heritage culture group (62).

On the other hand, bicultural identity is defined as knowing the language, lifestyle characteristics, and patterns of interpersonal behavior of two distinct cultural groups (67). It is positively affected well-being and negative effects acculturation stress (68). Besides, bicultural identification has a negative relationship with depression and anxiety (69). But it is thought that peer relationships might affect the development of identity and mental health. Phinney (63) suggested three stages model of ethnic identity development. First, unexamined ethnic identity individuals have unexamined positive or negative views of their ethnic group. Second, ethnic identity search individuals have begun a search into what it means to be a group member and achieved ethnic identity—individuals have explored their ethnic group membership. They are clear as to the meaning of ethnicity in their life. Acculturation perspective adapting to the host culture has been related to positive mental health (64, 65). These results consistently indicate that a higher level of ethnic identity is a protective factor for depression (22) and substance use (66).

As a result, the present research aims to examine the effect of peer relationships (peer attachment, social support), ethnic identity, and perceived discrimination on immigrant adolescents’ mental health. It is expected that peer attachment, ethnic identity, and social support might have buffering roles for mental health problems, whereas perceived discrimination might be a risk factor.

Based on the results of the studies mentioned above, the hypotheses of the study were as follows: (a) perceived discrimination will positively affect emotional problems and depression; (b) ethnic identity (both Syrian and Turkish) will negatively affect emotional problems and depression; (c) social support will negatively affect emotional problems, and depression (d) peer attachment (to both Syrian and Turkish peers) will negatively affect emotional problems and depression. To test these hypotheses, two hierarchical regression models were used.

## Method

### Procedure

Data for this study were collected from Syrian immigrant adolescents. Both ethical permissions for the study and the consent of school principals were obtained before the study. Parental consent form (in Arabic and Turkish) was sent to the homes, and none refused. All adolescents of the current sample had Arabic as their native language. Therefore, data were collected in Arabic. The researcher, together with an Arabic translator visited the schools for data collection. Participants were informed about the purpose of the study, the opportunity to refuse or discontinue participation at any time. They completed the questionnaires in a course hour.

### Participants

Syrian students constituted the biggest immigrant group in Turkish education system when compared to students with other national backgrounds (70). The sample included 226 Syrian immigrants (X^¯^_age_=13.31, SD=1.67, 70.8 % girls) aged between 10-18 who attended two different secondary schools for immigrant students only. The education was held in Arabic which is their home country language. 11.30% of them live separately from either their mother, father, or both. The minimum sample size was calculated using the formula suggested by Tabachnick and Fidell (71). According to this “50 + 8m” where “m” is the number of factors (in the present study m=6), so the minimum sample size is 106 for the current study.

Participants were living in a homogeneous social environment where the Syrian proportion is much higher resembling their home country and different from the rest of the region. For instance, all store signs are written in Arabic and schools provide education in Arabic for students. Immigrant adolescents have limited opportunity to meet a few Turkish peers in out of school environment. Thus, the participants seemed to have a limited chance for acculturation.

School principals and parents gave the approval for participation of the students. The study’s aim, confidentiality, opportunity to refuse or discontinue participation at any time were explained to the all participants. All students accepted participation and completed the research forms.

## Measures

*Demographic information*. Adolescents reported on their demographic information, including their gender, age, people they are living with, the number of siblings, Turkish proficiency level, the duration of living in Turkey, the duration of schooling in Turkey.

*Perceived discrimination.* Perceived discrimination was measured using a five-item scale, developed by International Comparative Study of Ethnocultural Youth (72). The items were answered on a 5-point Likert-type scale, ranging from strongly disagree (1) strongly agree (5). Sample item includes “I do not feel accepted by Turkish people”. Higher scores reflected higher levels of perceived discrimination.

*Social support.* Social support from classmates was measured using eight items (73). The items were answered 4-point Likert-type scale, ranging from totally disagree (1) totally agree (4). Sample item includes “I feel connected to the class”. The items assessed different aspects of emotional support, and higher scores reflected a higher level of emotional support.

*Depression.* Depression was measured using eight-item version of Center for Epidemiologic Studies Depression Scale (CES-D) (74). It is the short version of CES-D 20 (75). The items were answered 4-point Likert-type scale, ranging from (1) not at all to (5) often. Sample item includes “I felt like crying”. Higher scores reflected a higher level of depressive symptoms. The means, standard deviations, and bivariate correlations between the variables are presented in Table 1. Inspection of bivariate correlations showed that depression has significant correlations with emotional problems and perceived discriminations and had a negative correlation with Turkish peer attachment. Likewise, emotional problems had a significant positive correlation with depression and perceived discrimination and had a negative correlation with Turkish peer attachment. Besides, both Syrian and Turkish peer attachment had positive correlations with social support from classmates. Dramatically, Turkish ethnic identity had a positive correlation to both emotional problems and depression.

**TABLE 1.** Percentages of student samples in different demographic groups

	N	%
Gender		
Female	158	70.8
Male	68	29.2
Living time in Turkey		
Less than one year	19	8.5
Between 1-3 years	162	72.7
More than 3 years	42	18.8
Proficiency of Turkish		
Very well	66	32.2
Somewhat	112	13.2
Not at all	27	54.6

**TABLE 2.** Correlations and descriptive statistics of measured variables

	1	2	3	4	5	6	7	8	M	SD
1. Depression	1								19.06	6.30
2. Emotional problems	.34^**^	1							9.22	2.36
3. Social support	.11	.01	1						25.53	5.11
4. Peer attachment (Syrian)	.02	.06	.29^**^	1					13.41	2.49
5. Peer attachment (Turkish)	-.17*	-.15*	.05	.20^**^	1				11.76	5.64
6. Ethnic identity Turkish	-.05	-.14*	.15*	-.01	.11	1			7.03	2.81
7. Ethnic identity Syrian	-.02	.08	.02	.03	-.06	-.22^**^	1		10.94	1.88
8. Perceived discrimination	.27^**^	.27^**^	.10	-.08	-.14*	-.15*	.10	1	15.42	5.73

Note.*p<.05; **p<.01

*Predictors of depression*. The results of hierarchical multiple regression revealed that at step one, emotional problems contributed significantly to the regression model *F*_(1,224)_=28,951, p<.001 and accounted for 11% of the variation in depression of immigrants (*β*=.34, p<.001) was. Introducing both Turkish and Syrian peer attachment and social support from classmates in the second step explained 14% of the variation *F*_(4,221)_=8991, p<.001. But none of the variables were significant in the second step. Finally, the addition of ethnic identity and perceived discrimination to the regression model variables explained 17% variation in the immigrants’ depression. Increased perceived discrimination (*β*=.18, p<.001) was associated with increased depression level F_(7,218)_ = 6503, p <.01).

*Emotional problems.* Emotional problems were measured with the emotional symptoms subscale of Strengths and Difficulties Questionnaire (76). It consisted of 5 items. The items were answered on a 3-point Likert-type scale, ranging from not true (1) to certainly true (3). Sample item includes “I worry a lot”. Higher scores reflected a higher level of emotional problems.

*Peer attachment.*Peer attachment was measured with eight items; support from Turkish friends and co-ethnic friends (Arabic) includes four items (73, 77). The items were answered on a 4-point Likert-type scale, ranging from totally disagree (1) to totally agree (4). Sample items include “I feel connected to my friends”. Higher scores reflected greater social support and attachment.

*Ethnic identity.* Ethnic identity was measured with six items; three items for ethnic identity, three items for national identity (78). The items were answered on a 5-point Likert-type scale, ranging from strongly disagree (1) to strongly agree (5). Sample items include “I see myself as Turkish/Syrian”. Higher scores indicated greater levels of ethnic and national identity endorsement.

## Data analysis

To address the hypotheses, two hierarchical regression models were tested. In both models, the predictive roles of social and psychological factors were examined in separate steps. Before the regression analysis, preliminary analyses revealed no violations to the assumptions of homoscedasticity, normality, or linearity.

In the first model, where the depression was examined, the emotional problems were controlled in the first step due to the medium correlations between depression and emotional problems. Peer attachment and social support from classmates were entered in the second step. Ethnic identity and perceived discrimination were entered in the last step.

To predict emotional problems, the depression was controlled in the first step. Similar to the first model, peer attachment and social support from classmates were entered in the second step. In the last step, ethnic identity and perceived discrimination were entered into the regression equation.

## Results

### Descriptive results

The immigrant adolescent group consisted of 158 girls (70.8%), 68 boys (29.2%) whose ages from 10 to 17 (X^¯^ = 13.31, SD= 1.63). Table 1 provides demographic information of the sample.

The means, standard deviations, and bivariate correlations between the variables are presented in Table 1. Inspection of bivariate correlations showed that depression has significant correlations with emotional problems and perceived discriminations and had a negative correlation with Turkish peer attachment. Likewise, emotional problems had a significant positive correlation with depression and perceived discrimination and had a negative correlation with Turkish peer attachment. Besides both Syrian and Turkish peer attachment had positive correlations with social support from classmates. Dramatically, Turkish ethnic identity had a positive correlation to both emotional problems and depression.

### Predictors of depression

The results of hierarchical multiple regression revealed that at step one, emotional problems contributed significantly to the regression model *F*_(1,224)_=28,951, p<.001 and accounted for 11% of the variation in depression of immigrants (*β*=.34, p<.001) was. Introducing both Turkish and Syrian peer attachment and social support from classmates in the second step explained 14% of the variation *F*_(4,221)_=8991, p<.001. But none of the variables were significant in the second step. Finally, the addition of ethnic identity and perceived discrimination to the regression model variables explained 17% variation in the immigrants’ depression. Increased perceived discrimination (*β*=.18, p<.001) was associated with increased depression level F_(7,218)_ = 6503, p <.01).

## Predictors of emotional problems

The results of hierarchical multiple regression (Table 3) revealed that at stage one, depression (*β*=.34, p<.001) contributed significantly to the regression model *F*_(1,224)_=28,951, p<.001 and accounted for 11% variation in emotional problem of immigrants. Introducing Turkish and Syrian peer attachment and social support from classmate in the second step explained 14% of the variation *F*_(4,224)_=8376, p<.001. But none of the variables were not significant in the second step. Finally, the addition of ethnic identity and perceived discrimination to the regression model explained 17% variance in the immigrants’ emotional problems. Increased perceived discrimination (*β*=.18, p<.001) was associated with increased depression level *F*_(7,218)_=6653, p<.001.

**TABLE 3.** Results of hierarchical regression analyses regressing depression

Predictors	Step 1	Step 2	Step 3
Emotional problem	.34**	.32**	.28**
Peer attachment (Syrian)		-.01	.02
Peer attachment (Turkish)		-.12	-.11
Social support from classmates		.11	.09
Perceived discrimination			.18**
Ethnic identity (Syrian)			-.07
Ethnic identity (Turkish)			.00
Δ*R^2^*	.11**	.03	.03*
Total adjusted R^2^	.11**	.12**	.15**

Note.*p<.05; **p<.01

**TABLE 4.** Results of hierarchical regression analyses regressing emotional problems

Predictors	Step 1	Step 2	Step 3
Depression	.34**	.32**	.28**
Peer attachment (Syrian)		.09	.10
Peer attachment (Turkish)		-.12	-.09
Social support from classmates		-.04	-.05
Perceived discrimination			.18**
Ethnic identity (Syrian)			.05
Ethnic identity (Turkish)			-.07
Δ*R^2^*	.11**	.02	.04**
Total Adjusted R^2^	.11**	.12**	.15**

Note.**p<.01

## Discussion

In the current study, the effects of personal and social factors on immigrant adolescents’ mental health were investigated. The effects of peer relationships (attachment and social support), perceived discrimination, and ethnic identity on adolescents’ depression and emotional problems were investigated. The results showed that emotional problems and perceived discrimination predicted depression. Similarly, emotional problems were predicted by depression and perceived discrimination. But peer attachment, peer social support, and ethnic identity predicted neither depression nor emotional problems.

The positive effects of perceived discrimination on depression and emotional problems were as expected. Immigrants are faced with discrimination, such as name-calling and social exclusion (79). As a chronic stressor, perceived discrimination impacts immigrants’ mental health (29). In addition, perceived discrimination affects the acculturation process negatively (36-38). Ethnically homogenous environment offers feelings of safeness for immigrants. Immigrants, who continue their togetherness after migration can protect themselves from the host cultures’ uncontrollable attitudes and behaviors, improve their sense of group efficacy, and their psychological well-being (80) can maintain a sense of identity (81). Bagci and Canpolat (82) found perceived discrimination predicted the desire for contact for immigrant groups. Although Turkey is culturally “heterogenic”, researchers found group-based memberships is a critical issue (83), level of discrimination (84) and perceived discrimination (85) is high for minorities. It can be thought that the negative effect of perceived discrimination on mental health may be higher for immigrants living in a heterogeneous environment.

The second hypothesis assumed that ethnic identity would affect mental health problems negatively. It is known that a higher level of ethnic identity is related to mental health and the acculturation process (22, 66-68). Ethnic identity development is defined as a process related to feelings, opinions, thoughts, and actions related to ethnic group affiliation (62, 86). While positive identification processes are related to positive mental health outcomes (65), negative ones (such as marginalization and assimilation) were associated with negative mental health outcomes (21). Inconsistent results with the literature have been obtained for the second hypothesis. The correlation coefficients results showed that a negative correlation between emotional problems and Turkish ethnic identity. But there was no relationship between ethnic identity (both Turkish and Syrian) and depression. Ethnic identity development can occur as a result of exposure to cultural tools. As explained above, adolescents in this research have a little chance of exposure. Although they might be in ethnic identity search, as suggested by Phinney (63) they might not have a chance to search for and acquire an achieved ethnic identity. It should be supposed that more identity research is needed with a similar group.

The third and last hypotheses assumed that peer attachment and social support from classmates would negatively affect emotional problems and depression. Mental health is one of the main issues in the acculturation process. Immigrant adolescents have encountered difficulties causing negative mental health during the migration process (87, 88). Social support and peer attachment are related to positive mental health outcomes (22-25, 47, 48, 50, 52, 53). Results showed that in this research, peer support from classmates has no direct or indirect effect on immigrant adolescents’ mental health. Although Turkish peer attachment has negative correlations with depression and emotional problems, these relationships have not been found on regression analysis. According to this result, after controlling the effects of depression, peer attachment has not affected emotional problems. After controlling the effect of emotional problems, peer attachment has not affected depression. It is considered that social environments’ ethnic composition affects interpersonal relationships (58-61). Researches have shown that co-ethnic density might be a protective factor by lowering levels of acculturative stress (27, 57), identity threat (89), and bullying (61, 90). Conversely, other researches have emphasized more negative attitudes toward immigrants in the immigrant dense classroom (60, 91). When examining inconsistent results, the effects of the social environment on mental health consider characteristics, migration groups, contexts, and time to understand how acculturation is related to health and psychosocial outcomes (92). Participants have been attending ethnically homogenous classrooms with only Syrian students. They could meet with Turkish friends only out of school settings. Therefore, they have less chance to share experiences or emotions because of limited time and environment. It is known that acculturative processes (for instance, exposure to language, time spent in a new culture) relate to bicultural identity. Berry (20) suggests four components at the individual level for the acculturation process: *behavioral shifts* (such as language, food, dress), *acculturative stress* (resulting in disruptions in normal daily functioning), *psychological adaptation* (good mental health and a sense of well-being) and *sociocultural adaptation* (a set of social competencies). Participant adolescents might have low exposure to host country culture for the acculturation process suggested by Berry (20). They do not have to speak Turkish (*behavioral shifts)*, do not have outgroup friends into the school, and do not integrate in host culture education system and host culture (*sociocultural adaptation)*. In addition, they might have *acculturative stress* because of perceived discrimination. Therefore, the last component that is psychological adaptation is more negatively affected than other adolescents living in an immigrant-dense setting. More research is needed in a similar environment to test this finding.

Overall, when the results are evaluated depression and emotional problems affected each other with perceived discrimination. Social support, peer attachment, and ethnic identity did not predict mental health. Even if this result seems inconsistent with the literature, it should be considered that participants in this research represent a specific immigrant group.

Several limitations of the present study should be noted. First, the data were gathered in a specific area of one part of Turkey. Participants were more isolated than immigrant-dense. It will be beneficial to get the data from a similar group to generalize the future findings. Second, the self-report measurement was used. Although data will be more objective with peers, teachers, parents, or neighborhood, individuals’ own perceived competence within a valued domain is of most importance to their mental health (93). Finally, the inclusion of other social environments (parents, teachers, or neighborhood) would have added strength to the present study, but at the same time might be confounding variables. This study focused on the examination of peer-related variables. For future researches, the examination of variables from other social environments would be suggested.

## Clinical significance

It is known that immigrant adolescents faced different types of difficult life events in the migration process ranging from the parents’ loss to abuse. These life events are related to negative mental health outcomes. Immigrants who have negative experiences in the migration process try to adapt to a new culture; also, that can be a negative issue for them.

Acculturation influences the developmental process that defines an interaction between the organism and the context (6). Adolescents are in rapid changes in all aspects of development. Therefore, positive acculturation might be more critical for adolescents than adults. In addition, they may be a cultural intermediary between their family and host culture. As a result, their acculturation and mental health might affect life individual and social levels.

As researchers, we cannot know all the reasons for developing positive mental health in immigrant adolescents. Mental health may be related to issues at a different level. Besides the individuals’ characteristic, social structure (immigrant dense/sparse) or similarity of host and ethnic culture effects mental health. Thus, it would be crucial to have the research results evaluated by mental health workers and sociologists. They might examine together the characteristics of individuals and society to improve immigrants’ mental health.

## Disclosures

The author declares no conflicts of interest.
